# Oxygen-driven divergence of marine group II archaea reflected by transitions of superoxide dismutases

**DOI:** 10.1128/spectrum.02033-23

**Published:** 2023-12-04

**Authors:** Liping Qu, Meng Li, Fahui Gong, Lei He, Minchun Li, Chuanlun Zhang, Kedong Yin, Wei Xie

**Affiliations:** 1 School of Marine Sciences, Sun Yat-sen University, Zhuhai, China; 2 Department of Ocean Science & Engineering, Shenzhen Key Laboratory of Marine Archaea Geo-Omics, Southern University of Science and Technology, Shenzhen, China; 3 Southern Marine Science and Engineering Guangdong Laboratory (Zhuhai), Zhuhai, China; Hong Kong University of Science and Technology, Hong Kong, Hong Kong

**Keywords:** MGII, oxygen, superoxide dismutase, phylogenetics, molecular clock, evolution history

## Abstract

The superoxide anion radical (O^2•−^), the primary reactive oxygen species (ROS), is detrimental to cellular functions. Marine group II (MGII) archaea predominantly inhabit surface ocean environments that are rich in ROS; however, their defense mechanisms against ROS damage remain unknown. In this study, we integrated the analyses of archaeal 16S rRNA gene amplicons and metagenome-assemblage genomes of MGII archaea from the northern South China Sea and 250 other previously published MGII genomes to investigate their antioxidant mechanisms. The results show that the proportion of oxygen-promoted MGII operational taxonomic units in the iron/manganese superoxide dismutase (Fe/MnSOD) gene-containing group was significantly higher than that in the nickel SOD (NiSOD) group, suggesting that MGII members with Fe/MnSOD may have superior antioxidant capacity relative to members possessing NiSOD. Molecular clock analysis of MGII genomes revealed frequent species divergence correlating to the differentiation of Fe/MnSOD or NiSOD genes. The acquisition/loss of these genes in the MGII genomes was synchronized with the historical changes in atmospheric oxygen and oceanic metal element concentrations over the last 2.4 billion years, suggesting that variations in the antioxidant capacity of MGII archaea in response to changing oxygen levels may have driven their divergences over time.

**IMPORTANCE:**

Reactive oxygen species (ROS), including superoxide anion, is a series of substances that cause oxidative stress for all organisms. Marine group II (MGII) archaea are mainly live in the surface seawater and exposed to considerable ROS. Therefore, it is important to understand the antioxidant capacity of MGII. Our research found that Fe/Mn- superoxide dismutase (Fe/MnSOD) may be more suitable for MGII to resist oxidative damage, and the changes in oxygen concentrations and SOD metallic cofactors play an important role in the selection of SOD by the 17 clades of MGII, which in turn affects the species differentiation of MGII. Overall, this study provides insight into the co-evolutionary history of these uncultivated marine archaea with the earth system.

## INTRODUCTION

Oxygen is necessary for most life forms on earth because the energy release efficiency of aerobic respiration is much higher than that of anaerobic respiration; thus, it can support more complex life activities (1, 2). Currently, the percentage of oxygen in the earth’s atmosphere is about 21%, while it was less than 0.001% of today’s oxygen content during the Archaean Eon (3). Although the initial appearance of oxygen is still controversial, two major increases in atmospheric oxygen content have occurred. The first substantial increase occurred at 2.1 ~ 2.4 billion years ago (Gya), and is known as the Great Oxidation Event (GOE) (3). The second increase is known as the Neoproterozoic Oxygenation Event (NOE), which occurred at 0.8 ~ 0.55 Gya (3). Oxygen is important and fuels today’s biological diversity; however, exposure to excessive dissolved oxygen (DO) would damage microbial cells (4), mainly due to the presence of reactive oxygen species (ROS) (5). The superoxide anion (O^2•−^) is a form of ROS that can cause nucleic acid and protein damage to microorganisms (6). The elimination of O^2•−^ comprises two main steps. First, superoxide dismutase (SOD, EC 1.15.1.1) catalyzes the dismutation of O^2•−^ to H_2_O_2_, and then catalases and peroxidases catalyze the reduction of H_2_O_2_ to H_2_O. Thus far, three main types of SOD have been detected in the natural environment, namely copper-zinc-SOD (Cu-Zn-SOD) widespread in eukaryotes and a few Gram-negative bacteria (7, 8); nickel SOD (NiSOD) mainly found in Archaea and Bacteria (9); and iron/manganese SOD (Fe/MnSOD) found in both eukaryotes and prokaryotes (8, 10).

The first discovery of marine planktonic archaea in the surface and deep seawater was reported in 1992 (11, 12). Since then, four main groups have been described, specifically Marine Group I (MGI), Marine Group II (MGII), Marine Group III (MGIII), and Marine Group IV (MGIV) (12 – 15). Among which, MGII is classified as the order-level *Candidatus* Poseidoniales and contains two families: MGIIa (*Candidatus* Poseidonaceae) and MGIIb (*Candidatus* Thalassarchaeaceae). Different from MGI and MGIV, which are deep sea organisms (13, 15), MGII is widely distributed in global seawater (16) and have a higher abundance in the surface layer than in the deep sea (17, 18). The DO concentration could decrease from 4 mg/L to 9 mg/L in surface layer (0–200 m) to 1 ~ 2 mg/L in deeper layers (>200 m) at different sea areas (19 – 21), which means that MGII is at a higher risk of oxygen exposure. Metagenomic data has revealed that MGII are aerobic heterotrophs particularly capable of decomposing proteins, fatty acids, and polysaccharides (22 – 24). A higher relative abundance of MGII often occurs in particle-attached fractions composed of microalgae (24, 25) that concentrate DO around the cells of phytoplankton due to the production of oxygen (26). Our previous research found that an MGIIa from the Pearl River estuary might catalyze the reduction of H_2_O_2_ to H_2_O by acquiring the catalase gene via horizontal gene transfer (HGT) to cope with the high oxygen generated by the abundant phototrophs in this eutrophic area (27). However, the metabolic mechanism of MGII archaea resisting the stress of O^2•−^ is still largely unknown. In addition, unlike the well-studied evolutionary history of ammonia-oxidizing Thaumarchaeota archaea and their *amoA* genes (28, 29), the origin and differentiation of MGII archaea and their antioxidant genes are still elusive. The distribution pattern of SOD genes in the 17 clades (probably genus level) of MGII, the origin of MGII SODs, and their relationship with SODs of other origins (e.g., the sister cluster MGIII, other archaea, or even bacteria) are important and need to be understood.

In this study, we collected samples and corresponding environmental factors from the deep chlorophyll maximum (DCM) layer of the northern South China Sea (NSCS) to analyze the archaeal community alongside previously published 250 MGII reference genomes. Some abundant and DO-promoted MGII operational taxonomic units (OTUs) were identified through correlation and phylogenetic analyses. Functional annotation of the reference and newly reconstructed genomes demonstrated genomic evidence for the antioxidant role of SODs in MGII. Finally, HGT and molecular clock analyses were employed to investigate the origin and evolutionary history of the antioxidant capacity of MGII.

## RESULTS

**Fig 1:**
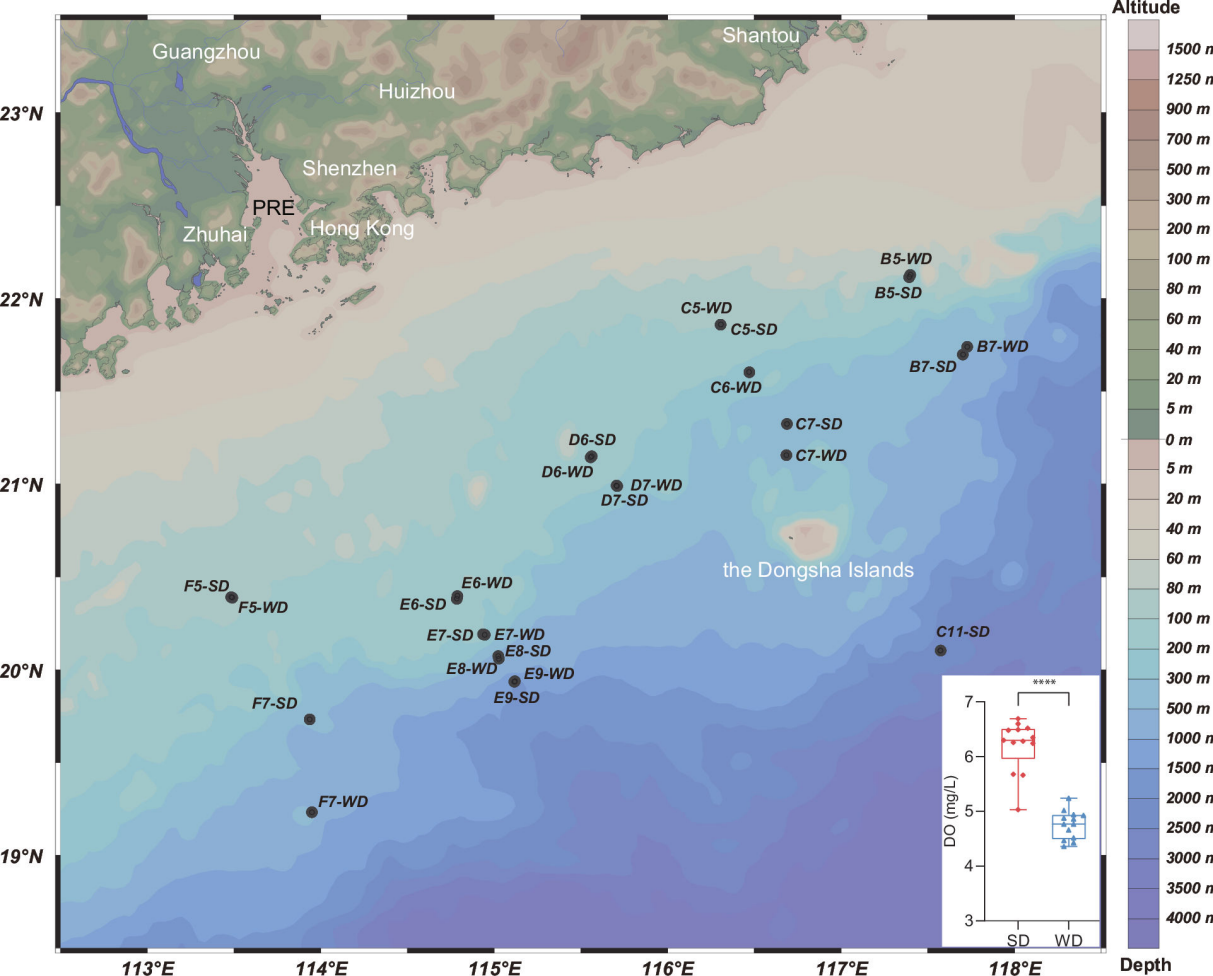
Sampling sites. Samples were obtained in the northern South China Sea in summer 2016 and winter 2018. Sampling sites are labeled by site ID and grouping mentioned in Table S1. SD indicates DCM samples in summer and WD indicates DCM samples in winter. The sample number of both groups is 13. A Student’s *t*-test was used to compare the DO concentrations between SD and WD samples. Altitude of land and depth of seawater are also shown. The map was created using Ocean Data View software.

In this study, seawater was collected from the DCM layer at 14 stations during summer and winter, generating a total of 26 samples (Fig. 1; Table S1). These samples were assigned to two groups, namely SD (DCM samples from the summer) and WD (DCM samples from the winter). Various environmental factors were measured *in situ*, including temperature, salinity, DO, and the concentrations of several nutrients (Table S1).

### The influence of environmental factors on individuals in the community

Considering that the dynamic changes of certain species may be concealed by the overall community, we investigated the influence of environmental factors on archaeal population at the OTU level. First, following the criterion described in the previous study (30), 39 OTUs with a relative abundance ≥1% in at least one sample were identified as abundant taxa (red IDs in Table S2). A total of 132 OTUs with a relative abundance >0.01% and <1% in at least one sample were considered as moderately abundant taxa, while the remaining 70 OTUs with relative abundance <0.01% in all samples were designated as rare taxa. Rare OTUs were excluded from the subsequent Spearman’s correlation and partial correlation analyses because small populations are more susceptible to random changes (e.g., genetic drift) as opposed to natural selection (e.g., environmental changes) (31); thus, their dynamic changes cannot truly reflect the effects of environmental factors. No MGII OTUs were identified as rare taxa.

**Fig 2:**
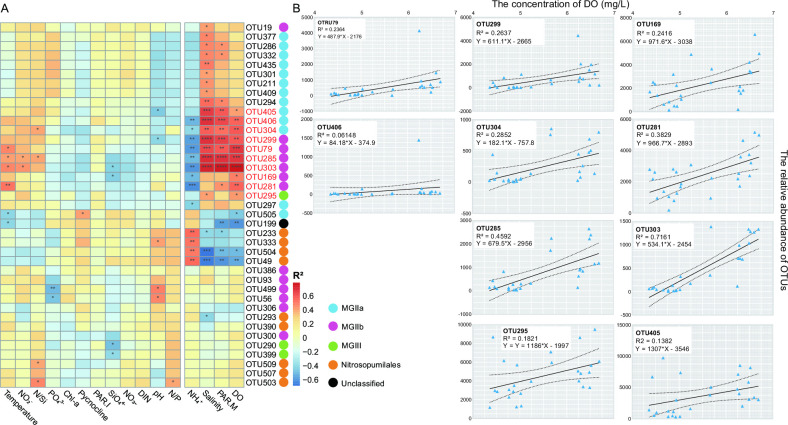
Relationship between environmental factors and the relative abundance of abundant OTUs. (**A**) Spearman’s correlations between environmental factors and the relative abundance of 39 abundant OTUs. Values of heatmap represent the correlation coefficient (*R*²). The “*,” “**,” “***,” and “****” represent *P* ≤ 0.05, *P* ≤ 0.01, *P* ≤ 0.001, and *P* ≤ 0.0001, respectively. Taxa of OTUs were indicated using solid circles with different colors. (**B**) Linear regression of the concentration of DO and the 10 DO-promoted OTUs. Blue triangles represent OTUs. Black dashed lines represent the 95% confidence intervals. Black solid line in each figure represents the linear regression results.

Redundancy analysis (RDA) of the archaeal community showed that only salinity had significant (*P* < 0.05) influence on the archaeal community (Fig. S1; Table S3). The greatest *R* ^2^ value was for the DO concentration, while the influence was not significant. Since the influence of environmental parameters on the structure of the entire archaea community is not significant (*P* = 0.351 for the RDA), Spearman’s correlation analysis was performed to investigate the influence of environmental factors on the relative abundance of the 39 abundant OTUs and the 132 moderate OTUs. In the DCM samples, the relative abundance of 10 abundant OTUs showed significant and positive correlations with the DO concentration and defined as “DO-promoted” OTUs (Fig. 2), six of which were also significantly promoted by salinity and the monthly average photosynthetically active radiation (PAR.M) but inhibited by C_NH4+_. Among the 10 OTUs whose relative abundance was promoted by DO, seven were affiliated with MGIIb, while OTU304 (no clade, *R* ^2^ = 0.60, *P* < 0.01) and OTU405 (clade-3, *R* ^2^ = 0.42, *P* < 0.05) were affiliated with MGIIa, and OTU295 (*R* ^2^ = 0.43, *P* < 0.05) was affiliated with MGIII (Fig. 2; Fig. S2). The seven MGIIb OTUs were affiliated with four clades, namely clade-9 (OTU169, *R* ^2^ = 0.49, *P* < 0.05), clade-10 (OTU285, *R* ^2^ = 0.69, *P* < 0.001, OTU303, *R* ^2^ = 0.79, *P* < 0.0001), clade-11 (OTU299, *R* ^2^ = 0.59, *P* < 0.01; OTU79, *R* ^2^ = 0.68, *P* < 0.001; OTU406, *R* ^2^ = 0.56, *P* < 0.01), and clade-14 (OTU281 *R* ^2^ = 0.57, *P* < 0.01). For the moderate OTUs, the relative abundance of 36 OTUs had significant and positive correlations with the DO concentration, among which 8 were MGIIa and 13 were MGIIb (Fig. S3). They also showed similar correlation patterns compared to the abundant OTUs, that most of them also promoted by salinity and PAR.M. However, C_NH4+_ only showed few correlations with the relative abundance of these OTUs. In addition, partial correlation was used to test the influence of the three parameters (salinity, PAR.M, and C_NH4+_) on the correlation between DO concentration and the relative abundance of OTUs. Amoung the 10 DO-promoted OTUs, five, three, and five of them still possessed significant and positive correlations with the DO concentration when controlling for salinity, PAR.M, and C_NH4+_, respectively (Table S4). The number of DO-promoted MGII OTUs increased to 19 (controlling for salinity), 11 (controlling for POR.M), and 20 (controlling for C_NH4+_) when all MGII OTUs were included in the partial correlation analysis (Table S5).

### Distribution of the oxygen tolerance potential in the MGII clades

The considerable positive correlation between MGII OTUs and DO made us curious about the metabolic reason implied in the genome. Therefore, to investigate the potential reasons for this oxygen tolerance differentiation, functional annotation was performed for 250 representative genomes of the 17 MGII clades (potential genus level) along with 13 outgroups used in a previous study (22), and four newly reconstructed genomes in this study were also included.

**Fig 3:**
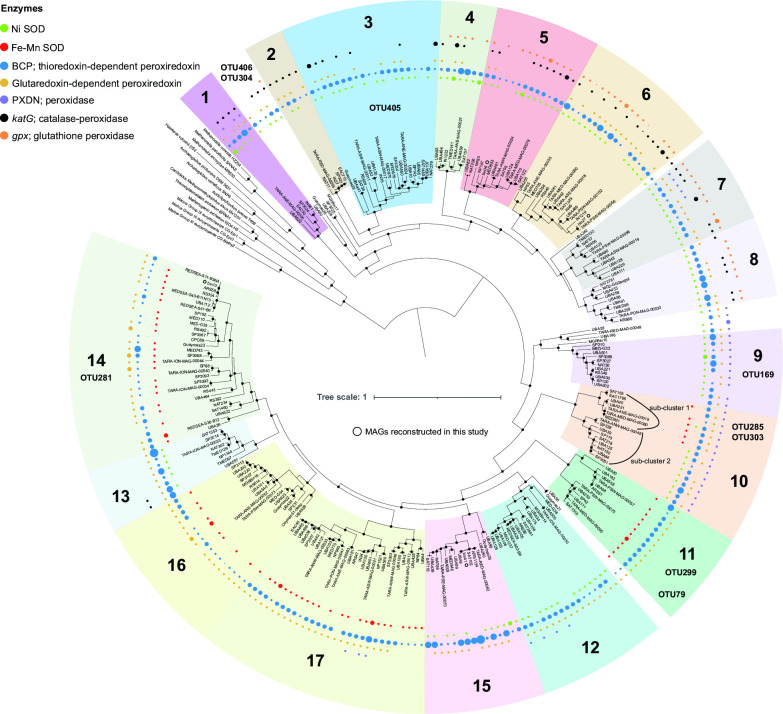
Phylogeny of MGII and the distribution patterns of antioxidant genes in different MGII subclades. The phylogenetic tree was constructed using 122 archaeal proteins. Reference genomes and subclades refer to previous study (20). MAGs newly reconstructed in this study are marked with white circles. Solid circles in the phylogeny indicate nodes with bootstrap values >95%. SOD genes were identified based on the Kyoto Encyclopedia of Genes and Genomes (KEGG) database. Solid circles around phylogenetic tree represent antioxidant genes identified in MGII genomes and different gene copy numbers are distinguished by the circle size. The identified MGII clades of nine DO-promoted and abundant MGII OTUs are shown.

Genes encoding two kinds of SOD were identified in those MGII metagenome-assemblage genomes (MAGs), namely NiSOD and Fe/MnSOD. Interestingly, most MGII archaea chose only one of them. All eight MGIIa clades (1 – 8) and four MGIIb clades (9, 12, 13, 15) only possessed NiSOD, but were missing Fe/MnSOD; genomes of four MGIIb clades (11, 14, 16, 17) only possessed Fe/MnSOD, but were missing NiSOD (Fig. 3). The sub-cluster 2 in clade-10 (MGIIb) only possessed NiSOD, while sub-cluster 1 mainly had Fe/MnSOD. Among the nine abundant DO-promoted MGII OTUs, four (OTU304 and OTU406, no clade; OTU405, clade-3; OTU169, clade-9) possessed NiSOD, while the remaining five (OTU285 and OTU303, sub-cluster 1, clade-10; OTU299 and OTU79, clade-11; OTU281, clade-14) were affiliated with a clade that mainly possessed Fe/MnSOD (Fig. 3). Among the 21 moderate DO-promoted MGII OTUs, 10 possessed NiSOD, 4 possessed Fe/MnSOD, and the remaining 7 could not assign to a certain MGIIb clade (Table S2). It should be noted that two sub-clusters in clade-10 possessed Fe/MnSOD and NiSOD (Fig. 3). The 16S rRNA gene identity of the three OTUs (OTU285, OTU303, and OTU235) and the two reference sequences (EAC1768 in sub-cluster 1 and SAT218 in sub-cluster 2) in clade-10 were both higher than 97% which is the threshold to distinguish different species (Table S6), but they all have higher BLAST scores with EAC1768 than with SAT218; therefore, we suggest they might be affiliated with sub-cluster 1. In addition, among the four newly reconstructed MAGs, NiSOD and Fe/MnSOD were identified in bin13 (clade-14) and bin27 (clade-6), respectively, while no SOD genes were found in bin36 (clade-25) and bin41 (clade-15), which may be due to the incompleteness of those MAGs.

The five DO-promoted abundant MGII OTUs within the Fe/MnSOD clade (FM-OTUs, 13 in total) and the four DO-promoted MGII OTUs within the NiSOD clade (Ni-OTUs, 38 in total) represented 38.5% and 10.5% of the total FM-OTUs and Ni-OTUs, respectively, in those samples (Table S2). These two values became 69.2% and 44.7% when DO-promoted moderate MGII OTUs were also included. Moreover, the determination coefficients (*R* ^2^) of the correlations between the five FM-OTUs and DO were also higher than those of the four Ni-OTUs to a certain degree (Fig. 2A and 3, ). These results indicate that MGII members with Fe/MnSOD gene may better adapt to oxidative stress than those with NiSOD gene in the DCM layer of the northern South China Sea. In addition, the distribution of five peroxiredoxins in the 17 MGII clades, which are another type of antioxidant enzyme, including thioredoxin-dependent and glutaredoxin-dependent peroxiredoxins, as well as the *PXDN*, *katG,* and *gpx* peroxidase-encoding genes, was investigated (Fig. 3). The thioredoxin-dependent peroxiredoxin was identified in most MGII genomes and was found as a multicopy gene in many members. In contrast, the genes *katG*/*gpx* and *PXDN* were mainly present in MGIIa and MGIIb, respectively.

### The superoxide dismutase protein in different organisms

Regular presence/absence patterns of the Fe/MnSOD and NiSOD genes in MGII were found in the previous analysis, and 202 archaeal genomes used in previous study (24) containing DPANN, TACK, Asgard, Euryarchaea, and *Candidatus* Thermoplasmatota (previously the Diaforarchaea group in Euryarchaea) were downloaded to further investigate this pattern in other archaea. Except two genomes of *Candidatus* Heimdallarchaeota (Asgard archaea), we found that most archaea only possessed Fe/MnSOD (Table S7).

**Fig 4:**
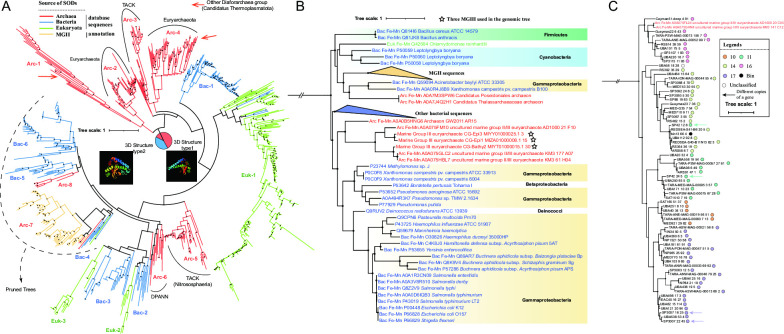
Horizontal gene transfer analysis of Fe-MnSOD. (**A**) Phylogeny of the Fe/MnSOD protein in Eukaryota, Bacteria, and Archaea. The phylogenetic tree was constructed using the protein sequences of Fe/MnSOD. SOD sequences identified in this study are highlighted in yellow and reference SOD sequences of Archaea, Bacteria, and Eukaryota are highlighted in red, blue, and green, respectively. Solid circles in the phylogeny indicate nodes with bootstrap values >70%. The distribution of two main 3D structures of Fe/MnSOD in different taxa were shown in the middle of the tree. (**B**) The pruned tree of the MGII cluster. Subclades of reference MGII genomes in Fig. 4 are marketed using solid circles in different colors. Multiple copies of SOD are marked using arrows. The root is cut off, which does not represent the real evolutionary distance. Solid circles in the phylogeny indicate nodes with bootstrap values >70%. (**C**) The pure tree of the sister cluster of MGII and MGIII sequences. The root is cut off, which does not represent the real evolutionary distance. Solid circles in the phylogeny indicate nodes with bootstrap values >70%.

Phylogenetic analyses were further performed using Fe/MnSOD protein sequences to distinguish their origin (Fig. 4 and Table S8). We found that the 750 Fe/MnSOD protein sequences formed several main phylogenetic groups, comprising three Eukaryota groups, six bacterial groups, and eight archaeal groups (Fig. 4A). Archaeal Fe/MnSOD was mainly identified in the Euryarchaeota and TACK groups, most of which are monophyletic groups except MGII and MGIII. The phylogenetic positions of the MGII and MGIII SOD proteins were very close, which is consistent with the species’ phylogeny. However, they were unique in that they clustered together with bacterial clades (Fig. 4A) rather than other archaeal sequences, which is a signal of HGT events. A pruned tree of the MGII and MGIII clusters and their sister clusters was further constructed, and we found that most members of sister clusters were Gammaproteobacteria (Fig. 4A and B). In previous annotation results, five clades (10, 11, 14, 16, 17) were found to possess Fe/MnSOD (Fig. 3 and 4C). The SOD cluster pattern at the protein level (Fig. 4) was consistent with the species phylogeny (Fig. 3), and the SOD sequences from these clades each formed a monophyletic group, except for one of the two SP42 SOD copies.

In addition, there were two main types of Fe/MnSODs with different 3D structures. The first type was conserved among four archaeal SOD groups (Arc-1, Arc-2, Arc-3, Arc-4), as well as Bac-1 and Euk-1, while the second type was found in the remaining SOD groups, namely Bac-2/3/4/5/6, Arc-5/6/7/8 and Eukaryota groups Euk-2/3 (Fig. 4A; Fig. S4). The structures between these two types are very similar, the only difference being that type 2 possesses an additional peptide chain between two long chains. An example is shown using yellow circles in Fig. S4.

**Fig 5:**
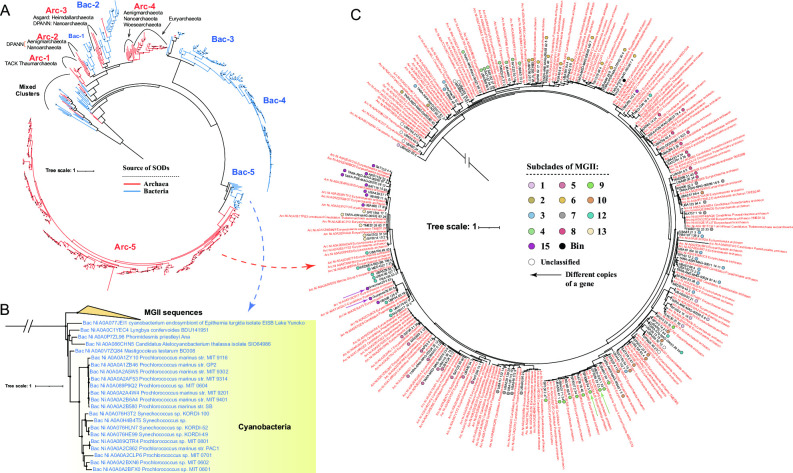
Horizontal gene transfer analysis of NiSOD. (**A**) Phylogeny of NiSOD proteins in Bacteria and Archaea. The phylogenetic tree was constructed using the protein sequences of NiSOD. SOD sequences of Archaea and Bacteria are highlighted in red and blue, respectively. Solid circles in the phylogeny indicate nodes with bootstrap values >70%. (**B**) The pure tree of the MGII cluster. Subclades of reference MGII genomes in Fig. 4 are marketed using solid circles in different colors. The root is cut off, which does not represent the real evolutionary distance. Solid circles in the phylogeny indicate nodes with bootstrap values >70%. (**C**) The pure tree of the sister cluster of MGII and MGIII sequences. The root is cut off, which does not represent the real evolutionary distance. Solid circles in the phylogeny indicate nodes with bootstrap values >70%.

We then performed a similar analysis for NiSOD. Although NiSOD was found in all archaea groups, including Asgard, DPANN, TACK, Euryarchaeota, and Thermoplasmatota, only 1 ~ 3 taxa within these groups possessed NiSOD (Fig. 5A and Table S7). Most MGII sequences were assigned to the Arc-5 group, with Cyanobacteria being the sister cluster of this huge group (Fig. 5B). Within this group, most members from the same MGII clade clustered together, but there were indeed some HGT signals that some clusters were separated into different positions within the phylogeny (Fig. 5C).

### Evolutionary history of MGII archaea and their superoxide dismutase genes

**Fig 6:**
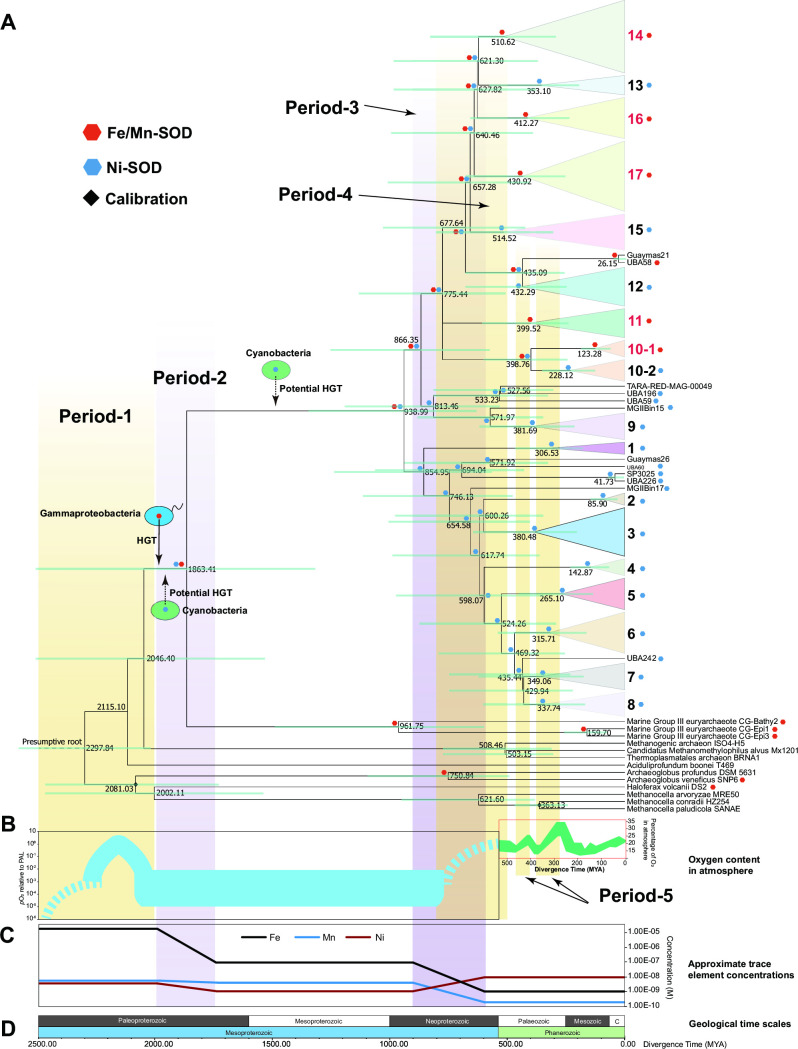
The co-evolution history of MGII Euryarchaea and associated SOD genes under the influence of environmental factors through time. (**A**) Evolution history of MGII archaea and diagrammatic sketch of SOD gene assignment process. Genes encoding Fe/MnSOD and NiSOD are shown using red and blue hexagons, respectively. Dashed arrows indicate assignment events of SOD genes. Nodes with calibration in the chronogram are indicated with red diamond. The green parallel line represents the error bar of the divergence time. (**B**) Evolution history of oxygen in atmosphere through time. The content dynamic change of oxygen in atmosphere during Mesoproterozoic was inferred from previous reference (3) and shown in the black rectangle. A more elaborate fluctuation during Phanerozoic was inferred from previous reference (29) and shown in the red rectangle. The content of oxygen is indicated using the atmospheric partial pressure of oxygen (pO_2_) relative to the present atmospheric level (PAL) and percentage of oxygen in the atmosphere in these two figures, respectively. (**C**) The content dynamic changes of some trace elements through time. The concentration of Fe, Mn, and Ni in the ocean were inferred from previous reference (30). (**D**) Geological time scales. The time scale was suitable for all panels in Fig. 5.

The evolutionary history of MGII was further inferred using the maximum likelihood method based on the molecular clock theory. The acquisition/loss history of the Fe/MnSOD and NiSOD genes through time was inferred based on the topology of the time tree (Fig. 5A) and genetic tree (Fig. 3), and the presence/absence patterns of the SOD genes annotated in the MGII clades. The percentage of oxygen content in the atmosphere (Fig. 6B) and the approximate trace element concentrations (Fig. 6C) over the geological time scale (Fig. 6D) were inferred from previous studies (3, 32, 33). The divergence time of MGII and MGIII was approximately 1,863.41 million years ago (Mya). It should be noted that the phylogenetic distance of Fe/MnSOD for these two archaeal groups was close; the position was far away from other archaeal sequences; and it clustered with Gammaproteobacteria (Fig. 4A). Furthermore, no HGT event was found within the MGII/MGIII cluster, which means that vertical gene transfer occurred after the gene acquirement (Fig. 4A). Therefore, we suggest that the Fe/MnSOD gene was obtained from Gammaproteobacteria via the common ancestor of MGII and MGIII between 2,046.40 ~ 1863.41 Mya, which represents the appearance and disappearance times, respectively, of the ancestor (Fig. 6A). The molecular clock suggests that this first HGT event occurred ~1863.41 Mya, which just follows the GOE (3). However, this ancestor of MGII and MGIII then experienced a period when the concentrations of iron and manganese decreased by 100 times and 5 times, respectively (Period-2 in Fig. 6).

The common ancestor of MGIIa and MGIIb appeared ~939 Mya, following a differentiation process that continued until recently (Fig. 6A). The appearance of MGIIb clades was mainly concentrated at 1,000 ~ 500 Mya, while the MGIIa clades have been continuously emerging during the more recent past, with clade-2 occurring even more recently at ~85.9 Mya. From about 900 ~ 600 Mya, the oceanic concentrations of Fe and Mn decreased from ~10^−7^ to ~10^−9^ M and ~6 × 10^−9^ to ~3 × 10^−10^ M, respectively, while that of Ni increased from ~10^−9^ to ~10^−8^ M (Period-3). It should be noted that most MGIIb differentiation processes occurred during this time (Fig. 6). In addition, the divergence of MGIIb was influenced by the second increase in the oxygen concentration during 800 ~ 500 Mya [the NOE (3); see Period-4 in Fig. 6A]. The divergence time of SOD in clade-12 and two sub-clusters in clade-10 were also consistent with Period-5, which consisted of two slight increases in the atmospheric oxygen content (Fig. 6A and B). For MGIIa, most divergence events also occurred during the increase in the oxygen, while the type of SOD did not change.

## DISCUSSION

### The DO concentration is a potential key factor controlling MGII distribution

The distribution of MGII is effected by multiple environmental parameters. For example, MGIIa and MGIIb have higher abundance in summer and winter, respectively (34), which reflected the influence of temperature to the MGII community. A previous canonical correspondence analysis suggested that the MGII community structure in many Tara Ocean samples was influenced by the concentration of oxygen (22). However, the specific impact of oxidative pressure on members of MGII (e.g., the 17 clades) and their adaptation mechanisms to oxidative pressure are still unclear. In this study, the *in situ* environmental parameters and archaeal community structure at the DCM of NSCS were combined to investigate their interactions. A Spearman’s correlation analysis based on 39 abundant OTUs and 132 moderate OTUs showed that mainly three environmental factors exhibited a clear promotion influence on some OTUs, namely salinity, PAR.M, and the DO concentration (Fig. 2; Fig. S3). A total of 10 abundant OTUs and 34 moderate OTUs had significant and positive correlations with the DO concentration of the DCM samples (Fig. 2), indicating a unique antioxidant potential among them. It should be noted that 9 of the 10 abundant OTUs including six MGIIb and three MGIIa OTUs, 21 of the 34 moderate OTUs including 8 MGIIa and 13 MGIIb OTUs, were affiliated with MGII. This suggests that the DO tolerance potential of MGII archaea, especially MGIIb, is better than other archaea living in the northern South China Sea. Indeed, some of these 30 MGII OTUs also exhibited a significant positive correlation with PAR.M (Fig. 2; Fig. S3) and partial correlation analysis also could not thoroughly exclude the impact of PAR.M (Table S4 and S5); however, a suitable PAR promotes algal growth, which will further promote oxygen production. Therefore, the influence of PAR on archaea should be similar to that of DO.

Significant correlations were also identified between salinity/NH_4_^+^ concentration and the relative abundance of MGII OTUs. However, no matter which of these two factors was controlled, most significant correlations between DO concentration and the relative abundance of abundant and moderate MGII OTUs still exist (Table S4 and S5). Additionally, previous metagenomic analyses have indicated that MGII archaea do not have ammonia-oxidizing metabolic potential (16, 22, 25) , and no ammonia-oxidizing genes were identified in any of the MGII genomes in this study. Thus, we also suggest that MGII do not have ammonia-oxidizing potential and the NH_4_^+^ concentration should not be a key influencing factor for MGII archaea.

In summary, the dynamic change in the relative abundance of these 30 MGII OTUs was most likely due to the DO concentration.

### The characteristics of superoxide dismutase of MGII

The *in situ* association between the relative abundance of some MGII OTUs and the DO concentration (Fig. 2A) was identified in previous analyses. Functional annotation and phylogenetic analysis of the antioxidant genes were further performed to investigate the principle of the MGII antioxidant capacity.

Previous studies found that most bacteria with Fe/MnSOD, such as Actinobacteria, Gammaproteobacteria, and Bacteroidetes, tend to lack NiSOD and vice versa (35, 36). In this study, both SODs were identified in MGII, and we found that most MGII clades, except clade-10, only possessed one SOD type (Fig. 3). However, even for the clade-10 exception, this rule was confirmed at a deeper phylogenetic level, in that each sub-cluster possessed either Fe/MnSOD or NiSOD, but not both. The relative abundances of nine abundant MGII OTUs had a significant and positive correlation with the DO concentration (Fig. 2), five of which were FM-OTUs and the remaining four were Ni-OTUs (Fig. 3). It should be noted that the *R* ^2^ of the correlations between the five FM-OTUs and DO were higher than those of the four Ni-OTUs (Fig. 2A and 3; Fig. S3). The higher correlation suggests that Fe/MnSOD could help MGII members better adapt to high oxygen conditions. We further investigated all 69 MGII OTUs to uncover more evidence for this conjecture. Overall, 38 OTUs possessed NiSOD and 13 OTUs possessed Fe/MnSOD, whereas the SOD type was unknown for the remaining 18 OTUs (Table S2). The five abundant FM-OTUs and four abundant Ni-OTUs represented 38.5% and 10.5% of the total FM-OTUs and Ni-OTUs, respectively. Moreover, the percentage increased to 69.2% and 44.7% for the FM-OTUs and Ni-OTUs, respectively, if all DO-promoted MGII OTUs were considered. In summary, the number of MGII OTUs with Fe/MnSOD was higher than OTUs with NiSOD, and the former have higher *R* ^2^ with the DO concentration; both results suggested that Fe/MnSOD might have a better antioxidant capacity than NiSOD within the MGII population. In addition, the increase of metal cofactor concentration may also help for the growth of MGII, as the synthesis of SOD will have sufficient materials. Previous studies suggested that the concentrations of dissolved Fe (dFe), Mn (dMn), and Ni (dNi) are about 0.2 nM (37, 38), 3.7 nM (39), and 2 ~ 3 nM (37) in the surface seawater at the NSCS. The concentration of dNi is close to dMn and obviously higher than dFe. It means that the MGII members containing NiSOD should grow similarly to or even better than members containing Fe/MnSOD if high metal cofactor concentration can indeed promote the growth of MGII. However, our results showed that the actual situation is exactly the opposite. There were more MGII OTUs containing Fe/MnSOD than NiSOD and the former has a higher *R* ^2^ value (Fig. 2; Table S2), which means that the MGII members containing Fe/MnSOD grow better than members containing NiSOD even under the lower metal concentration (dFe). The more likely explanation is the potential different antioxidant capacity of MGII containing Fe/MnSOD or NiSOD, rather than the improvement of high concentration of metal cofactors to the assembly of SOD.

Spectroscopy evidence has shown that the active sites are very similar within the Fe/MnSOD family (Fe-, Mn-, or Fe/MnSOD) but different from the NiSOD family (40). In this study, we also found that the predicted 3D structure of Fe/MnSOD was different from that of NiSOD in the MGII archaea (Fig. S4 and S5). However, due to the uncultivable characteristics of MGII members, we have no direct evidence to demonstrate whether the structural difference between MGII-type Fe/MnSOD and MGII-type NiSOD caused differentiation of the DO adaption potential of the 17 MGII clades. The structural differentiation of proteins from different SOD groups (Fig. S3 and S4) was very small, suggesting that differentiation occurred mainly with respect to the amino acid composition of the protein sequences rather than affecting the advanced folding structure.

### The horizontal gene transfer of MGII SOD genes

HGT is common in prokaryote, including MGII archaea. Previous phylogenetic analysis of fosmid sequences suggested that there are frequent HGT events that occurred between bacteria and MGII (41). This pattern was subsequently confirmed at genomic level that 29.7% of the genes in the pangenome of group II/III Euryarchaea were mediated by HGT events. It should be noted that HGT could occur both early and at a recent time (42). In this study, we found that MGII SOD genes were transferred from bacteria through HGT events in the early evolution stages of MGII based on phylogenetic and molecular clock analysis (Fig. 4-6). At the protein level, most archaeal Fe/MnSOD formed several single clusters in the phylogeny, while only MGII and MGIII sequences clustered with bacterial SODs (Fig. 4A). In the pruned tree, most of the bacterial SODs were associated with Gammaproteobacteria (Fig. 4B). The phylogenetic distance of SODs from MGII and MGIII was close, which was consistent with the species tree, but far away from other archaeal sequences. This is a clear signal that HGT might have mediated the transfer of Fe/MnSOD genes from these bacteria to the common ancestor of MGII and MGIII. All MGII SOD proteins formed a monophyletic group, which suggests that there were no HGT events after the MGII ancestors obtained genes encoding Fe/MnSOD. Furthermore, the Fe/MnSOD protein sequences from the same MGII clade clustered together (Fig. 4C), suggesting that this gene was inherited vertically and conserved at a deeper phylogenetic level in MGII.

A previous study mainly identified NiSOD in Nanoarchaeota and Thermoplasmatota (9). In this study, we also found a few NiSOD sequences in Thaumarchaeota, Aenigmarchaeota, Heimdallarchaeota, and Woesearchaeota (Fig. 5A and Table S8). Although there were many reference sequences marked as “Euryarchaeota” in the UniProt database (Fig. 5C), it should be noted that these references were submitted before MGII was assigned as Thermoplasmatota and that many MGII sequences have not been further identified but only marked as “Euryarchaeota.” Another reason is that such frequent HGT events should not occur in each phylogenetic cluster of MGII (Fig. 5C). Since a previous global analysis has also suggested that Thermoplasmatota was the main contributor of NiSOD (9), we argue that most NiSOD came from MGII rather than Euryarchaeota and other Thermoplasmatota. Indeed, we found evidence for the rare occurrence of NiSOD in the genomes of other archaea (Table S8). In summary, most archaea possessed Fe/MnSOD, and it was unique for such wide distribution of both Fe/MnSOD and NiSOD in MGII. This may be because the main habitat of MGII is surface seawater, which causes higher oxidative stress, and more selectable OD enzymes may help them better adapt to this environment. In addition, phylogenetic analysis suggests that NiSOD genes of MGII were also transformed from bacteria via HGT, and that the contributor was Cyanobacteria. However, we cannot exclude the possibility that NiSOD genes flowed from MGII to Cyanobacteria. Like the Fe/MnSOD HGT event of MGII, there were also no HGT events of NiSOD between bacteria and MGII subclades (Fig. 5A); thus, we argue that the HGT event involving NiSOD occurred between the ancestors of these two species (Fig. 6A).

### The evolutionary history of MGII

Organisms are always affected by the environment and evolve to adapt to their environment. To understand the evolutionary history of the MGII SOD genes under the influence of the environment, we focused on some environmental factors, including the percentage of oxygen in the atmosphere, and the concentration of several metallic elements, specifically Fe, Mn, and Ni, which make up the metal core of SOD enzymes. In the classical two-step model, the oxygen content increased during the GOE about 2,300 Mya (43), while a recent study suggests that the oxygen concentration continuously changed from 2.5 to 2.0 Mya (3). Paleontological sequencing research found that the origin of SOD may be earlier than the GOE due to some local increases in oxygen concentration (44). Since the MGII/MGIII SOD sequences had a close evolution distance and the same donor species (Fig. 4A and B), we suggest that the Fe/MnSOD genes of MGII and MGIII might have been transformed from Gammaproteobacteria to their common ancestor about 2,046.40 ~ 1,863.41 Mya (Fig. 6A), which was very close to the GOE (Period-1 in Fig. 6A). This indicates the adaptive evolution of MGII mediated by HGT in response to the increasing pressure of oxidation. As so far, the evolution history of MGII and their closely related species still have controversy. In a recent study, Lu et al. suggested an earlier divergence time of MGII and MGIII than our results, which was ~2,718 Mya based on an untreated phylogenetic tree (45). However, it should be noted that the divergence time in the treated tree (the divergence time became ~2,361 Mya after 20% heterogeneous sites were removed) of Lu’s research and the potential time region suggested in our research (Fig. 6) were both close to the time region of the first GOE. Therefore, we suggested that the high oxygen concentration may be one reason that caused the divergence of MGII and MGIII archaea, which could be reflected by the differentiation of SOD genes.

Different from the clear history of Fe/MnSOD genes, there were two potential scenarios for the evolution of MGII NiSOD, and the main uncertainty lies in the acquired time of NiSOD (Fig. 6A). The first thing that can be determined is that the ancestors of MGII must have this gene, because both MGIIa and MGIIb possessed the NiSOD gene. However, we could not rule out the other possibility that the ancestor of MGII and MGIII obtained both NiSOD and Fe/MnSOD, then MGIII only inherited an Fe/MnSOD gene. Therefore, in the first scenario, the transfer of NiSOD occurred at the same time as Fe/MnSOD between Cyanobacteria and the ancestor of MGII/MGIII, and the divergence of MGII and MGIII happened under the changing metal content (the increase in Fe and Mn, see Fig. 6C). Alternatively, in the second scenario, the ancestor of MGII obtained the NiSOD gene from Cyanobacteria via HGT between 1,863.41 and 938.99 Mya, and differentiation of MGIIa and MGIIb followed. The first scenario is more likely, because the ancestor of MGII/MGIII suffered a decrease in the Fe/Mn elements, which was a strong selection pressure for the loss of NiSOD.

After the HGT events of the two SOD genes and the differentiation of MGII and MGIII, a more complex evolutionary process occurred at about 1,000 ~ 500 Mya. In particular, frequent divergences of MGII occurred intensively from about 950 ~ 250 Mya (Fig. 6A). After the divergence of MGIIa and MGIIb and the assignment event of Fe/MnSOD and NiSOD genes at about 938.99 Mya, constant differentiation of the MGIIb ancestor occurred, assigning these two genes to different subsequent species. Most clades of MGIIb emerged at 1,000 ~ 500 Mya, with most inheriting only one of these genes from their ancestors. This SOD gene assignment process also occurred in recent years. For example, the divergence of clade-12 and the two subclades of clade-10 occurred at about 400 Mya. In contrast, the differentiation processes of MGIIa were evenly distributed over a longer time range, from about 1,000 Mya to 85.9 Mya which was the occurrence time of clade-2.

There were four important environmental changes during this period, specifically the decrease in Fe and Mn elements (about 900 ~ 600 Mya), the increase in the Ni element (about 900 ~ 600 Mya, Period-3), and the increase in the oxygen content (about 800 ~ 500 Mya, Period-4). The lower Fe and Mn had a negative effect on the biosynthesis of Fe- or Mn- SOD, while the increase of Ni was a signal for MGII to use NiSOD, causing a tendency for archaea to lose the gene encoding Fe/MnSOD. All evidence suggests that the changes in SODs and the associated environmental factors indeed contributed to the evolution of MGII archaea.

The time points for population segmentation and functional gene assignments were consistent with the changes in metallic elements related to SOD and the oxygen content, indicating that evolution of the antioxidant capacity dominated the MGII evolution. In addition, a previous study (35) and our results both suggest that these two types of SOD genes were not retained in the genomes at the same time, which may be because of the common gene loss strategy that occurs during the evolution process of microorganisms (46).

### Conclusion

Our results demonstrated significant positive correlations between the relative abundance of nine abundant MGII OTUs and the DO concentration in the DCM layer of the northern South China Sea, suggesting adaptation of these MGII to the high oxygen environment. Fe/MnSOD and NiSOD were found to separately distribute in different MGII clades, and MGII members with the Fe/MnSOD might have a better antioxidant capacity than members with the NiSOD. The MGII SODs were unique, in that all the genes were sourced from bacteria. We found that the Fe/MnSOD genes might have been obtained from Gammaproteobacteria by the common ancestor of MGII and MGIII, and these genes continue to be inherited vertically until today. Alternatively, the MGII NiSOD was transferred from Cyanobacteria. The frequent assignment events of Fe/MnSOD and NiSOD genes may be a strategy for coping with the changing oxygen and metallic element concentrations in the ocean over time. The evidence from both the ecologic and phylogenomic analyses supports the crucial role of the antioxidant capacity in the divergence of MGII, revealing the evolutionary history of these universally abundant, but poorly understood marine archaeal lineages.

## MATERIALS AND METHODS

### Sampling and measurements of environmental parameters

In the summer of 2016 and the winter of 2018, seawater was collected and environmental parameters were measured at the DCM layer (40–100 m) of the northern South China Sea. Briefly, a bottle of seawater was collected from different depths at different stations. During sampling, environmental parameters including temperature, salinity, depth, pH, and *in situ* photosynthetically active radiation (PAR.I) were measured at each layer using the SeaBird SBE 19+ conductivity, temperature, and depth (SeaBird SBE 19plus CTD) system (SeaBird, Bellevue, WA, USA), which contains two extra sensors including the SBE43 dissolved oxygen sensor (SeaBird, Bellevue, WA, USA) and the Seapoint turbidity meter (Seapoint Sensors, Brentwood, NH, USA). All sensors were calibrated before the cruise. The concentrations of nutrients (NO_3_^-^, NO_2_^-^, NH_4_^+^, PO_4_^3 -^, SiO_4_^2-^) in the sampled seawater were measured by spectrophotometry using a mobile nutrient analyzer (Seal, Germany). The monthly average PARs (PAR.M) were downloaded from the NASA’s OceanColor Web (https://oceancolor.gsfc.nasa.gov/). After sampling, 2 L of seawater of each layer was filtered by a six-way seawater filtration device (Pall, USA) containing a cellulose acetate membrane (48 mm diameter) with pore size of 0.22 µm. The filter process was performed on board under the condition of pressure less than 0.03 MPa. After filtering, the membrane was stored in a 50 mL centrifuge tube with no reagent, and cryopreserved at −80°C directly. For the chlorophyll-a, 1 L seawater was filtered using the same method except that the cellulose acetate membrane was replaced with the GF/F glass microfiber membrane (Whatman, UK). The membrane was then put in a 15 mL tube and 10 mL cooled acetone solution (90%, w/w) was added in the tube. After ultrasonication for 10 minutes, the tube was stored overnight at 4 °C. Next, the supernate was transferred to the other 15 mL tube and centrifuged at 4000 revolutions per minute (rpm) and 4 °C for 10 minutes. Finally, the concentration of chlorophyII-a was measured using the 10-AU Fluorometer (Turner Designs, USA). All steps of the chlorophyII-a extration was finished in dark.

### DNA extraction and 16S rRNA gene sequencing

DNA extraction was performed using the FastDNA spin kit for soil (MP biomedical, USA), and the DNA concentration was measured using the Nanodrop 2000c spectrophotometer (ThermoFisher, USA) and it was between 5.23 ng/µL and 20.79 ng/µL (Table S1). The V4–V5 hypervariable region of the archaeal 16S rRNA gene was amplified using the primers 524F10extF (5´-TGYCAGCCGCCGCGGTAA-3′) and Arch958RmodR (5′-YCCGGCGTTGAVTCCAATT-3′) (47, 48) , and the following amplification system: The PCR mixtures contained 4 µL of 5× TransStart FastPfu buffer, 2 µL of dNTPs (2.5 mM), 0.8 µL of each primer (5 µM), 0.4 µL of TransStart FastPfu DNA Polymerase, 10 ng of template DNA, and, finally, ddH_2_O up to 20 µL. The amplicons were paired-end sequenced using an Illumina Novaseq 6000 platform (Illumina, San Diego, USA) according to the standard protocols by Magigene Biotechnology Co. Ltd. (Guangzhou, China).

### Analysis of 16S rRNA gene high-throughput sequencing data

Quality control of the original sequencing sequence was performed using fastp v0.20.0 (49) software with default parameters. Splicing was performed using the Fast Length Adjustment of SHort reads v1.2.7 (50) software based on the following criteria: (i) bases with a tail mass value of less than 20 in reads were filtered; (ii) if the average mass value in a 50 bp window is less than 20, the back-end bases from the window were cut off; (iii) reads with the tail mass value of less than 50 bp after quality control or containing N bases were removed; (iv) according to the overlapping relationship between reads, paired reads were spliced into a sequence, and the minimum overlap length was 10 bp; (v) the maximum allowable mismatch ratio of overlap region of splicing sequence was 0.2, and the non-conforming sequences were screened; (vi) the samples were distinguished according to the barcodes and primers at both ends of the sequence, and the sequence direction was adjusted; (vii) the allowable mismatch number of barcodes was 0 and the maximum primer mismatch number was 2.

OTUs with 97% similarity cutoff (51, 52) were clustered using UPARSE v7.1 (51), and chimeric sequences were identified and removed at the same time. The taxonomy of the representative sequence of each OTU was analyzed using the Ribosomal Database Project (RDP) Classifier v2.2 (53) against the silva database release 132 (54), and the confidence threshold was 0.7.

### Quality control, assembly, binning, and functional annotation of metagenomic data

Parts of DNA extracts of the 0.2 µm filter membrane from 4 m and 100 m of site E9 (Fig. 1) in winter were used to perform the whole genome amplifications using the REPLI-g Single Cell kits (QIAGEN, Germany) following the manufacturer’s protocol. Amplifications were paired-end sequenced on Illumina Hiseq Xten (Illumina Inc., San Diego, CA, USA) at Majorbio Bio-Pharm Technology Co., Ltd. (Shanghai, China) using Illumina Novaseq 6000 platform (Illumina, San Diego, USA) according to the standard protocols by Majorbio Bio-Pharm Technology Co. Ltd. (Shanghai, China). The raw reads from those metagenome sequencing were used to generate clean reads by removing adaptor sequences, trimming, and removing low-quality reads (reads with N bases, a minimum length threshold of 50 bp and a minimum quality threshold of 20) using the fastp software (49). Contigs with a length being or over 300 bp were selected as the final assembling result. CONCOCT (55), MaxBin2 (56), and metaBAT2 (57) with default parameters were used parallelly to produce MAGs. A total of 17 MAGs having >50% completion and <10% contamination were identified from those contigs. Four out of the 17 MAGs were annotated as MGII archaea with GTDB-Tk v1.3.0 (58) with default parameters, while the others were Bacteria. Protein-coding genes of all MGII genomes were identified using prodigal (59) with default parameters, and their functions were further annotated using the online Kyoto Encyclopedia of Genes and Genomes (KEGG) (60) servers with default parameters.

### Taxonomy assignment analysis of OTUs and bins

Since the resolution of the RDP classifier was not enough to affiliate OTUs to the family (MGIIa and MGIIb) or genus levels (the 17 clades) for MGII archaea, further taxa of all MGII OTUs and the four newly reconstructed MGII MAGs from the northern South China Sea obtained in this study were investigated by phylogenic and sequence identity analyses based on the 16S rRNA gene. Phylogenetic analysis based on 122 archaeal single-copy genes was additionally employed for the four MAGs. The ascription of nine DO-promoting MGII OTUs at the genus level was shown in Fig. 3.

The phylogenetic analyses of all MGII OTUs identified using RDP classifier and four newly reconstructed MGII MAGs were performed separately. Briefly, The 16S rRNA genes of 250 reference MGII genomes (22) were first extracted using Barranp v0.9 (https://github.com/tseemann/barrnap) with “--kingdom arc” parameter which could apply for the archaeal database in this software. These 16S rRNA gene sequences and two outgroup sequences of MGIII were aligned using MAFFT v7.310 (61) and trimmed using trimAl v1.4.rev15 (62) with default parameters. Then, a maximum likelihood (ML) tree was constructed using IQTREE v2.1.3 (63) software with the Modelfinder (64) for model selection based on the “-m MFP” option, and a total of 1,000 ultrafast bootstrap replicates were sampled based on the “-B 1000” option to assess the robustness of the phylogeny (65). The phylogenetic tree was visualized using iTOL v5.6 (66). Next, OTUs were affiliated with one of the MGII clades (22) based on the phylogeny. This assignment was confirmed by the paired comparison of OTU sequences and the 16S rRNA gene sequences of reference MGII genomes by using blastn (67). For four newly reconstruct genomes (MAGs), a phylogenetic tree based on 122 archaeal proteins was constructed. Briefly, the 122 archaeal protein sequences (68) of the four MAGs, the 250 reference MAGs mentioned above, and 13 genomes as outgroups were extracted using the “identify” command in the GTDB-TK v1.5.1 (58) software. Next, the extracted sequences of each protein were aligned and all alignments were linked together to generate a super multiple sequence alignment (MSA) for each genome using the “align” command. The MSA was applied to construct the phylogenetic tree using FastTree2 v2.1.11 (69) with “-gamma -lg” options. The phylogenetic tree was visualized using iTOL v5.6 (66).

All MAGs used in the previous genomic tree and two genomes of DPANN archaea downloaded from the National Center for Biotechnology Information database (GCA_003086475.1 and GCA_000008085.1) were applied for the species divergence time analysis using the “RelTime-ML” method (70) in MEGA v11 (71). A new ML phylogenetic tree was constructed using the same method mentioned above. These two DPANN genomes were considered as the new outgroup, and the initial phylogeny was rooted here. Same as the phylogenetic tree, the Le Gascuel model (72) was also selected as the substitution model in this analysis, and a chronogram was constructed using MEGA. The molecular dating was calibrated during the construction process based on the range of divergence time between *Methanocella conradii* and *Methanocella paludicola* (1,087.6 ~ 3,759.8 Mya) and between *Archaeoglobus profundus* and *Methanocella arvoryzae* (186.9 ~ 508.2 Mya). The reference range of divergence time was queried from the TimeTree of Life website (73).

### Identification of HGT events, and 3D structure prediction of SOD protein sequences

Two kinds of SOD were identified in MGII genomes. Phylogenetic analysis was performed to explain the origin of these enzymes and identify if there are some HGT events between MGII and other organisms. Archaea, Bacteria, and Eukaryota domains were analyzed together in this phylogeny. We downloaded 250 Fe/MnSOD protein sequences from the UniProt database (74) for each domain, then a total of 750 reference sequences were mixed with Fe/MnSOD sequences identified in the 254 MGII genomes (250 reference genomes and four newly reconstructed MAGs) and used for subsequent analysis. All protein sequences were aligned using Muscle v5.1 (75) and then trimmed using trimAl v1.4.rev15 (62) with default parameters. Finally, the phylogenetic tree was constructed using FastTree2 v2.1.11 (69) with “-gamma -lg” options and visualized using iTOL v5.6 (66). A similar analysis was also performed for NiSOD, but only Bacteria and Archaea were analyzed because NiSOD was not characterized for Eukaryota and no available sequences were found in the UniProt database. The 3D structure prediction of representative sequences of Fe/MnSOD and NiSOD was performed using web server Phyre v2.0 (76) with default parameters.

### Statistical analysis

The Student’s *t*-test of the DO concentration was performed using GraphPad Prism version 9 (https://www.graphpad.com/). The RDA was performed using the “vegan” v2.6–4 (77) and the “ggplot2” v3.3.5 (78) packages in R (79). Before the RDA, the data of environmental parameters were first logarithmically transformed based on the natural constant *e* and the data of OTU numbers were Hellinger transformed. The Spearman’s correlation analysis was performed using the “corr.test” function in the R package “psych” v2.2.5 (80).

## Data Availability

The raw data of high-throughput sequencing of 16S rRNA genes and metagenomic sequencing are available at NCBI under the accession number PRJNA815943.

## SUPPLEMENTAL MATERIAL

Supplemental figures spectrum.02033-23-s0001.pdf Figures S1 to S5.

Table S1 spectrum.02033-23-s0002.xlsx Information and environmental parameters of sampling sites.

Table S2 spectrum.02033-23-s0003.xlsx Archaeal community structure at OTU level.

Table S3 spectrum.02033-23-s0004.xlsx Results of RDA analysis in Fig. S1.

Table S4 spectrum.02033-23-s0005.xlsx Partial correlation analysis for the 39 abundant OTUs.

Table S5 spectrum.02033-23-s0006.xlsx Partial correlation analysis for all MGII OTUs.

Table S6 spectrum.02033-23-s0007.xlsx BLAST results of 16S rRNA genes of 39 abundant OTUs and 250 reference MGII MAGs.

Table S7 spectrum.02033-23-s0008.xlsx Annotation results of Fe/MnSOD and NiSOD in 202 reference archaeal genomes.

Table S8 spectrum.02033-23-s0009.xlsx Summary of SOD protein sequences used in the HGT analysis.

ASM does not own the copyrights to Supplemental Material that may be linked to, or accessed through, an article. The authors have granted ASM a non-exclusive, world-wide license to publish the Supplemental Material files. Please contact the corresponding author directly for reuse.
