# Supplementary material for: Oxygen-driven divergence of marine group II archaea reflected by transitions of superoxide dismutases
Source: Microbiol Spectr. 2023 Dec 4;12(1):e02033-23. doi: 10.1128/spectrum.02033-23 (PMC10783094; doi:10.1128/spectrum.02033-23)
Supplement: Supplemental figures — Figures S1 to S5. [file spectrum.02033-23-s0001.pdf]

Supplementary Materials for

Oxygen-driven divergence of marine group II archaea reflected by transitions of superoxide dismutases

Liping Qu *et al*

Corresponding author: Wei Xie, Email: xiewei9@mail.sysu.edu.cn.

This PDF file includes:

Supplementary Figure S1 to S5.

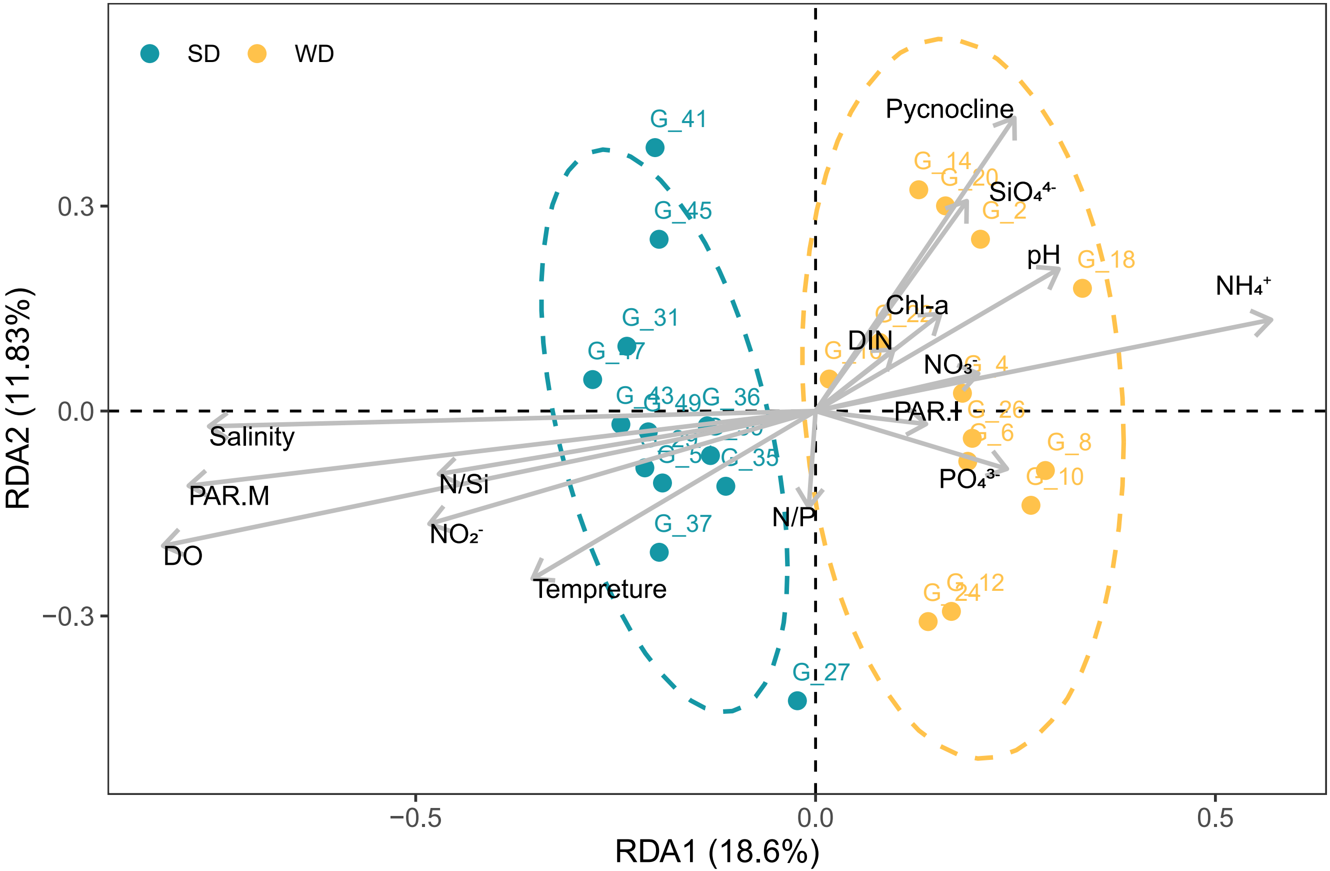

Fig. S1. RDA ordination diagrams. PAR.M: average PAR; PAR.I: in situ PAR; Pycnocline: The depth of pycnocline. Green and yellow circles represent SD and WD samples, respectively.

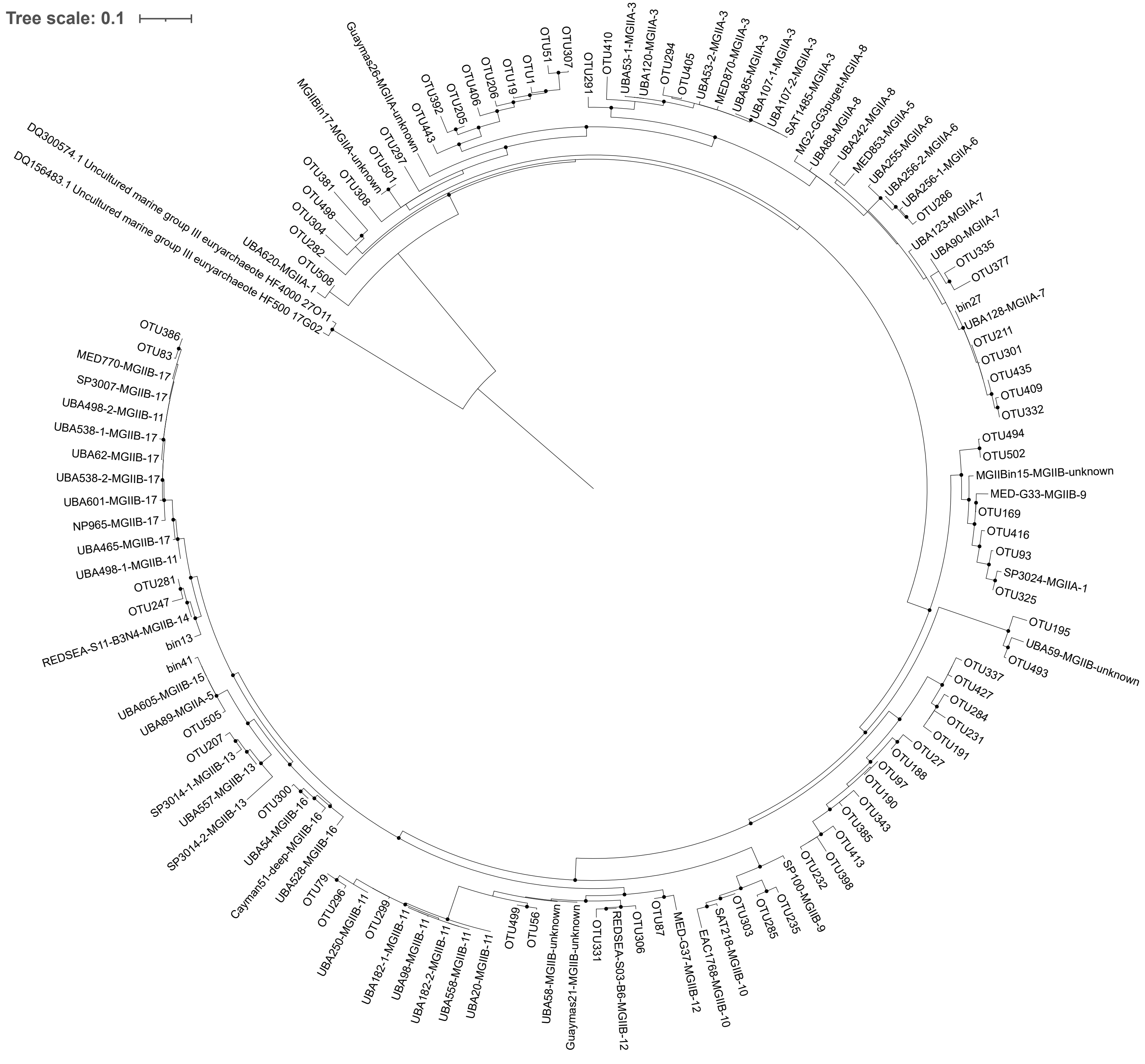

Fig. S2. Maximum-likelihood phylogenetic tree shows the taxonomic assignment of MGII OTUs. This phylogenetic tree was constructed using the reference 16S rRNA genes extracted from 250 reference MGII MAGs and the 69 MGII OTUs identified in this study. Remaining reference MAGs with no available 16S rRNA genes are not used to construct the phylogenetic tree. Solid circles in the phylogeny indicate nodes with bootstrap values > 70%.

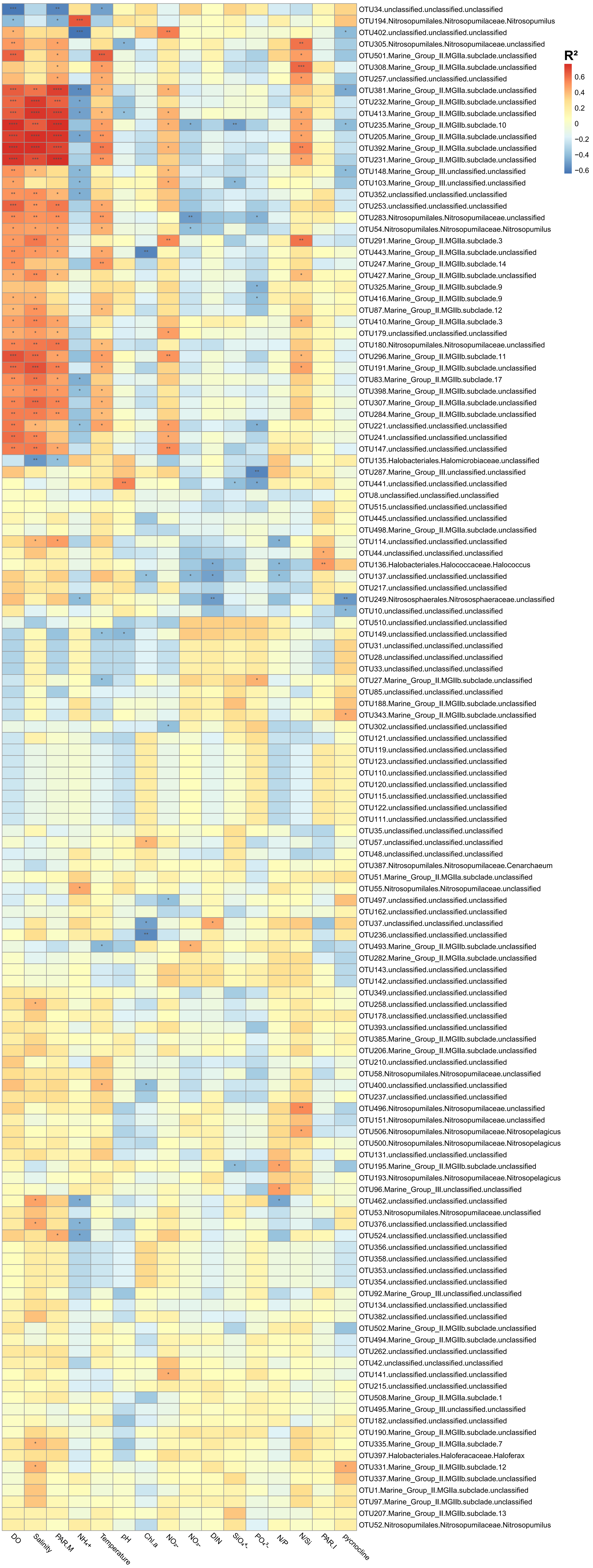

Fig. S3. Spearman's correlations between environmental factors and the relative abundance of the 132 moderate OTUs. Values of heatmap represent the correlation coefficient (R^2). The '*', '**', '***' and '****' represent $p \le 0.05$, $p \le 0.01$, $p \le 0.001$, and $p \le 0.0001$, respectively. OTU IDs consist by the OTU ID, order, family and genus name.

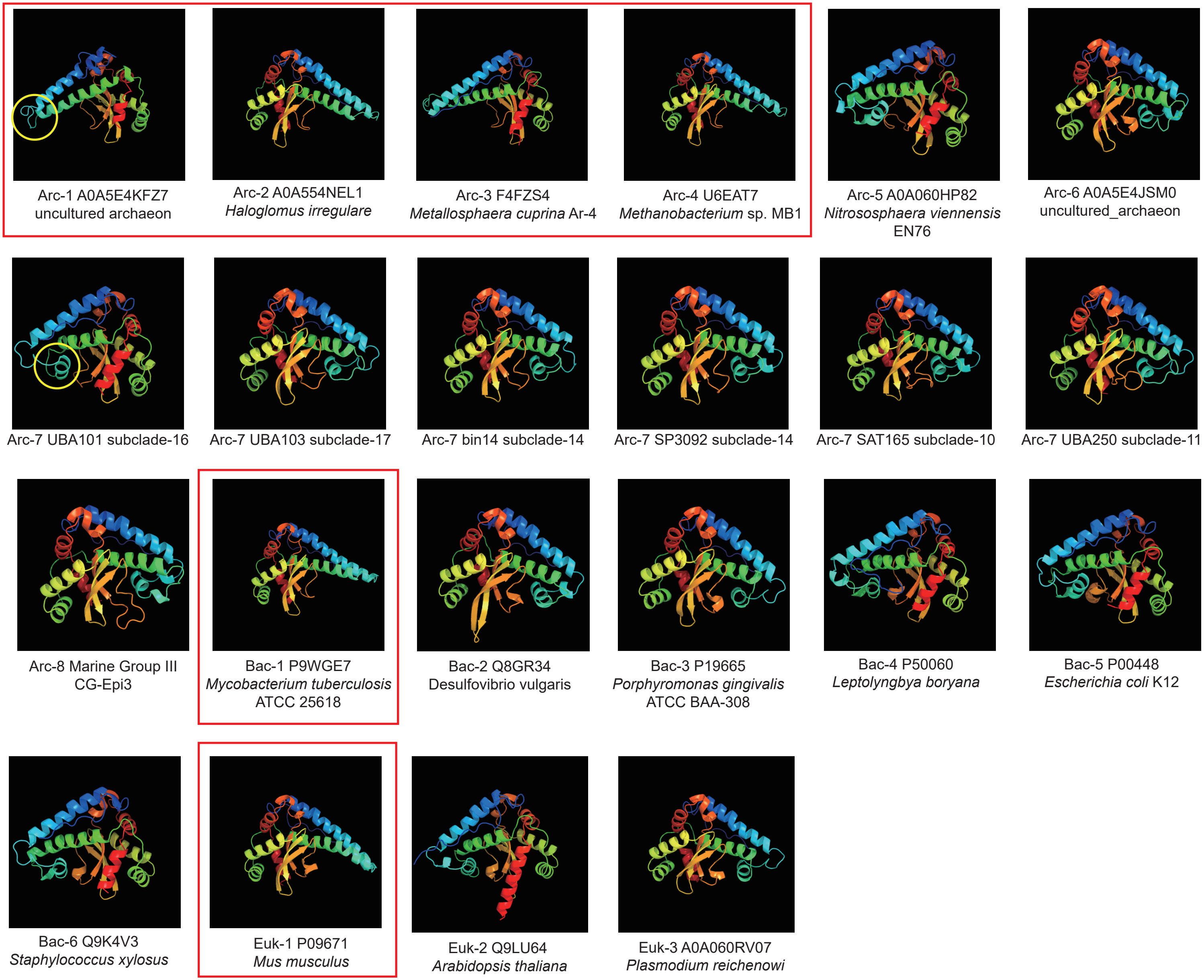

Fig. S4. 3D-structure of representative Fe/MnSOD proteins. The description below each figure consists of SOD-group ID in Fig. 4A, the protein ID in the UniProt database, and the Latin name of the source species of the SOD sequence. The description of MGII cluster consists with SOD-group ID, genome ID, and subclades ID of MGII. Pictures belong to the 3D structure type1 of Fe/MnSOD were marked with red rectangles.

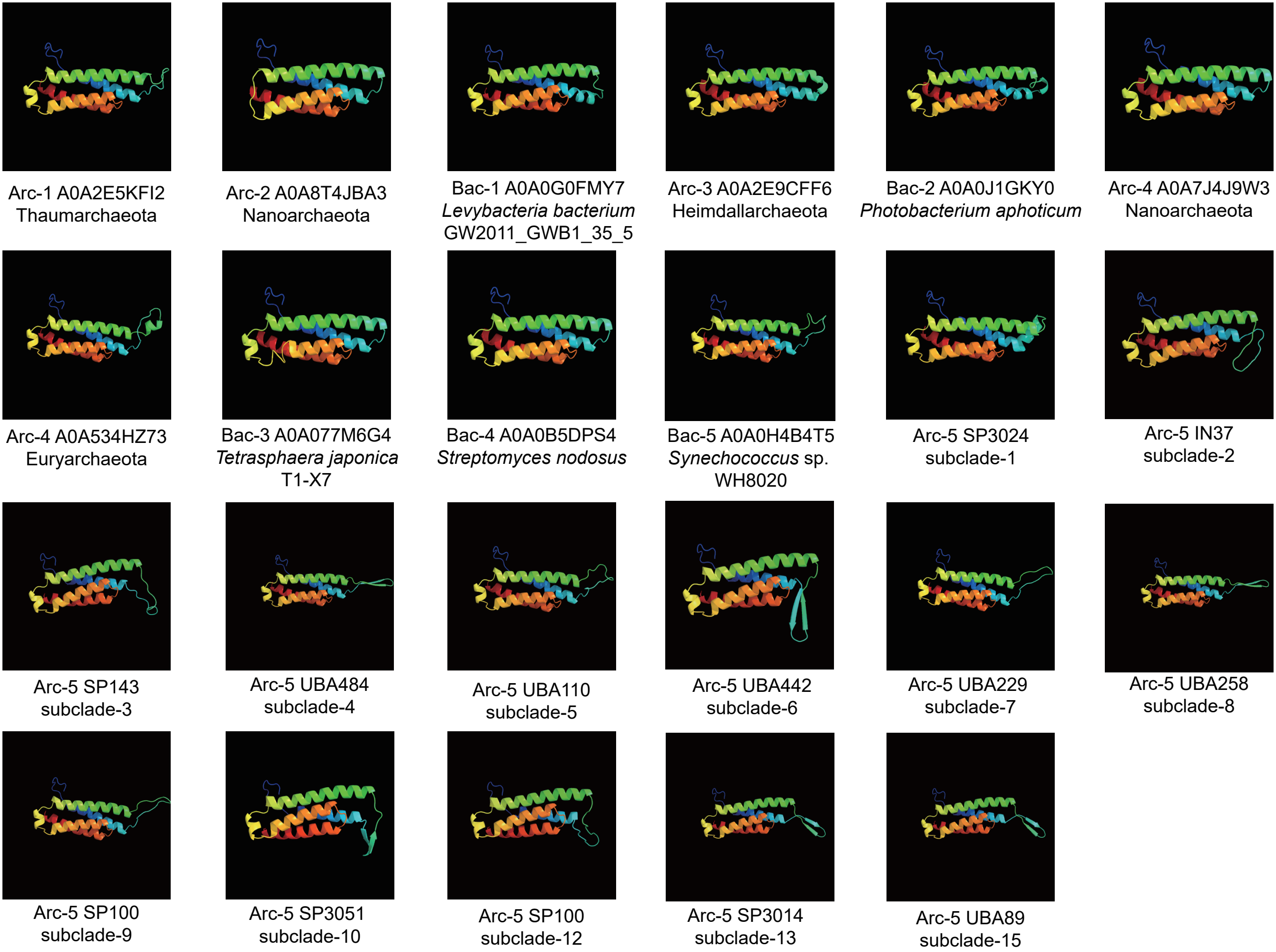

Fig. S5. 3D-structure of representative NiSOD proteins. The description below each figure consists of SOD-group ID in Fig. 5A, the protein ID in the UniProt database, and the Latin name of the source species of the SOD sequence. The description of MGII cluster consists with SOD-group ID, genome ID, and subclades ID of MGII.
